# Sertraline Reduces IL-1β and TNF-α mRNA Expression and Overcomes Their Rise Induced by Seizures in the Rat Hippocampus

**DOI:** 10.1371/journal.pone.0111665

**Published:** 2014-11-03

**Authors:** María Sitges, Carlos D. Gómez, Blanca I. Aldana

**Affiliations:** Departamento de Biología Celular y Fisiología, Instituto de Investigaciones Biomédicas, Universidad Nacional Autónoma de México, Distrito Federal, México; University of Utah, United States of America

## Abstract

We recently discovered that the antidepressant sertraline is an effective inhibitor of hippocampus presynaptic Na^+^ channel permeability *in* *vitro* and of tonic-clonic seizures in animals *in* *vivo*. Several studies indicate that the pro-inflammatory cytokines in the central nervous system are increased in epilepsy and depression. On the other hand inhibition of Na^+^ channels has been shown to decrease pro-inflammatory cytokines in microglia. Therefore, the possibility that sertraline could overcome the rise in pro-inflammatory cytokine expression induced by seizures has been investigated. For this purpose, IL-1β and TNF-α mRNA expression was determined by RT-PCR in the hippocampus of rats administered once, or for seven consecutive days with sertraline at a low dose (0.75 mg/kg). The effect of sertraline at doses within the range of 0.75 to 25 mg/kg on the increase in IL-1β and TNF-α mRNA expression accompanying generalized tonic-clonic seizures, and increase in the pro-inflammatory cytokines expression induced by lipopolysaccharide was also investigated. We found that under basal conditions, a single 0.75 mg/kg sertraline dose decreased IL-1β mRNA expression, and also TNF-α expression after repeated doses. The increase in IL-1β and TNF-α expression induced by the convulsive agents and by the inoculation of lipopolysaccharide in the hippocampus was markedly reduced by sertraline also. Present results indicate that a reduction of brain inflammatory processes may contribute to the anti-seizure sertraline action, and that sertraline can be safely and successfully used at low doses to treat depression in epileptic patients.

## Introduction

Psychiatric disorders, and particularly depression, are known as frequent co-morbidities in patients with epilepsy [1]–[7]. Interestingly, evidence that inflammatory processes take place in both illnesses, namely depression and epilepsy, has been provided. For instance, the hypothesis that inflammation plays an important role in depression, initially suggested by some pioneer studies [8]–[10], was further supported by various meta-analyses indicating that some pro-inflammatory cytokines are increased in patients with major depressive disorders [11]–[13]. The involvement of brain pro-inflammatory cytokines in seizure generation and maintenance, as well as in the establishment of chronic epileptic focuses, has been amply documented [14]–[20].

Voltage sensitive Na^+^ channels are particularly involved in exacerbating neuronal excitability during seizures. Thus, among the most effective of all anti-epileptic drugs are those capable of decreasing voltage sensitive Na^+^ channel permeability. In hippocampal isolated nerve endings, sertraline, a drug broadly prescribed for the treatment of depression [21], in addition to its action on the 5-HT transporter, resulted in a potent and effective inhibitor of voltage sensitive Na^+^ channel permeability [22]. Additionally, in lipopolysaccharide (LPS) stimulated microglia, sertraline inhibited the production of the pro-inflammatory cytokine tumor necrosis factor-α (TNF-α) [23]. In mixed glial cell cultures the Na^+^ channel blocker, tetrodotoxin, as well as the classic anti-epileptic drug, phenytoin, whose mechanism of action involves inhibition of Na^+^ channels, reduced the secretion of the pro-inflammatory cytokines interleukin-1beta (IL-1β) and TNF-α induced by lipopolysaccharide [24]. Therefore, the possible *in* *vivo* action of sertraline on the cerebral expression of those inflammatory markers was tested in the hippocampus, which is a highly epileptogenic brain structure. Recently we found that generalized tonic-clonic seizures induced by the pro-convulsive agents pentylenetetrazole (PTZ), and 4-aminopyrydine (4-AP), increased the expression of IL-1β and TNF-α in the rat hippocampus (unpublished results), and that sertraline prevented seizures and the epileptiform EEG activity induced by those pro-convulsive agents [25]. Therefore, we also measured the effect of sertraline on the increase in those pro-inflammatory cytokines induced by seizures.

## Materials and Methods

### Source of Materials

4-aminopyridine (4-AP) and lipopolysaccharide (LPS, *Escherichia coli*, serotype 0127:B8) were obtained from Sigma-Aldrich (St. Louis, MO), pentylenetetrazole (PTZ) was obtained from MP Biochemicals Inc. (Aurora, Ohio) and sertraline was kindly donated by Psicofarma S.A. de C.V. (México).

### Animal Groups

The present study was carried out in strict accordance with the recommendations in the *Guide for the Care and Use of Laboratory Animals of the Official Mexican Standard* (NOM-062-ZOO-1999). The protocol was approved by the Committee on the Ethics of Animal Experiments of the Instituto de Investigaciones Biomédicas, Universidad Nacional Autónoma de México. All efforts were made to minimize animal suffering.

The 59 male Wistar rats (294±2.5 g initial weight) included in the present study were divided in the 12 groups summarized in Table 1. Briefly, the control Group 1 (G1) was administered either with saline, or with the vehicle used to dissolve sertraline, which consisted of 70% saline and 30% DMSO. No statistical difference in the pro-inflammatory cytokines expression in animals injected with saline or the sertraline vehicle was found. Group 2 (G2) received a single injection of sertraline at a dose of 0.75 mg/kg, and Group 4 (G4) a daily injection of 0.75 mg/kg sertraline for one week. Group 3 (G3) was administered with a daily injection of the sertraline vehicle for one week and used as the control for G4. Group 5 (G5), was administered once with saline followed by 4-AP; Group 6 (G6), was administered once with 0.75 mg/kg sertraline followed by 4-AP, Group 7 (G7) with 0.75 mg/kg sertraline daily for one week, followed by 4-AP, Group 8 (G8) with saline followed by PTZ; Group 9 (G9) with sertraline at a dose of 2.5 mg/kg followed by PTZ, and Group 10 (G10) with 25 mg/kg sertraline followed by PTZ.

**Table 1:** Experimental Animal Groups.

Groups	One Injection				Seven Injections		One Injection		
	Vehicle	Sertraline			Vehicle	Sertraline	4-AP	PTZ	LPS
		0.75 mg/kg	2.5 mg/kg	25 mg/kg		0.75 mg/kg			
G1	√								
G2		√							
G3					√				
G4						√			
G5	√						√		
G6		√					√		
G7						√	√		
G8	√							√	
G9			√					√	
G10				√				√	
G11	√								√
G12			√						√

*√, indicates injection(s) of the substance(s) specified on the headlines.*

*4-AP = 2.5 mg/kg 4-aminopyridine; PTZ = 50 mg/kg pentylenetetrazole; LPS = 100 µg/kg lipopolysaccharide.*

4-AP and PTZ were both dissolved in saline and injected *i.p.* at convulsive doses of 2.5 mg/kg and 50 mg/kg, respectively. After injections of 4-AP the animals were observed for 1 h, and after the injection of PTZ for 30 min before euthanasia. These two time points of euthanasia were defined on the basis of our previous study [25].

The effect of sertraline on the changes induced by LPS in the hippocampal IL-1β and TNF-α mRNA expression was tested in two additional groups: Group 11 (G11) that was injected with saline before LPS (100 µg/kg *i.p.*) inoculation, and Group 12 (G12) that was injected with 2.5 mg/kg sertraline before LPS. The animals in these two groups were sacrificed 1 h following LPS.

**Figure 1:**
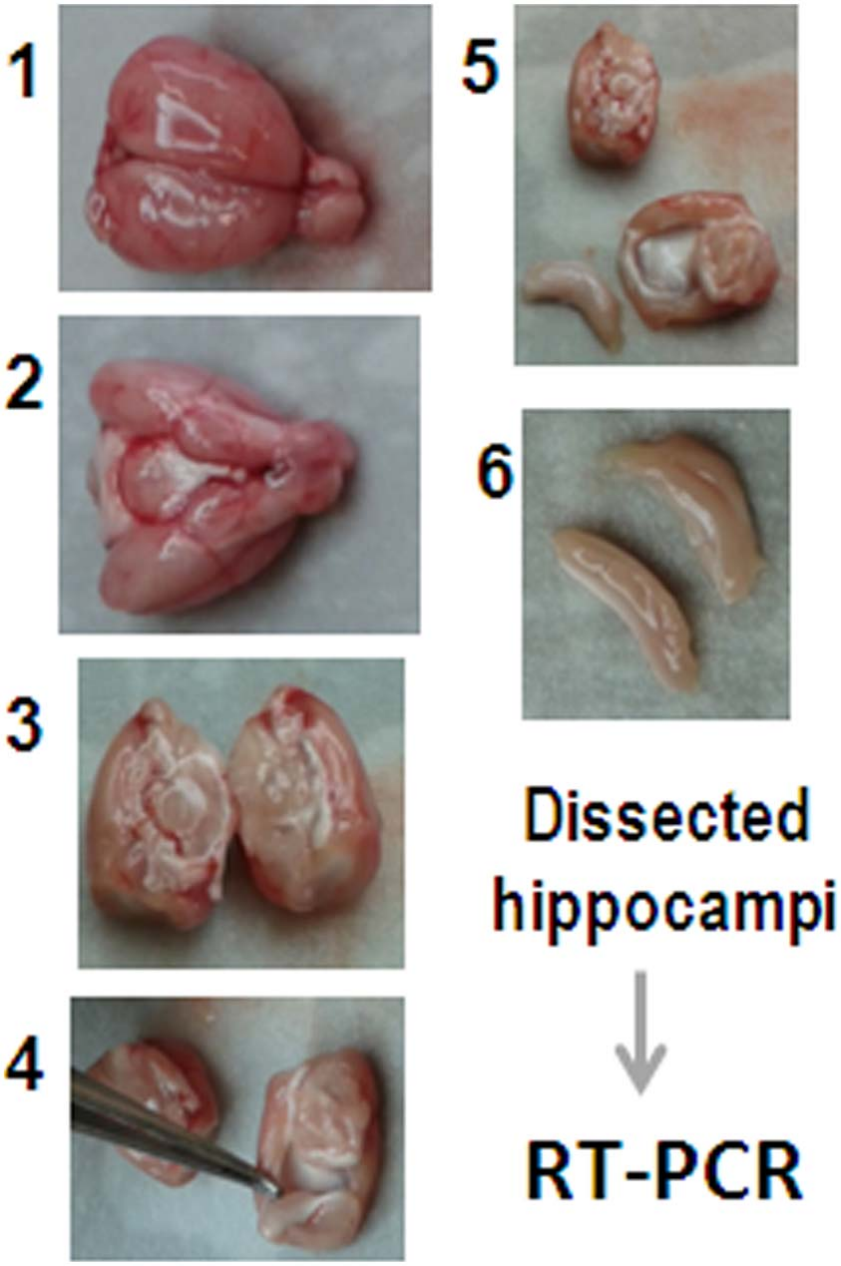
Hippocampus Dissection. Following decapitation, the brain was exposed and the hippocampus immediately dissected as shown. The whole process takes less than 3 min.

In an effort to minimize stress, the animals of all Groups were rapidly decapitated and their hippocampi immediately dissected (see Fig. 1) and frozen.

Sertraline was always administered 4 h before 4-AP, PTZ or LPS. This particular time was chosen because sertraline, after 4 h was reported to reach a high concentration in rat brain tissue [26].

### RNA Extraction

For isolating the hippocampal RNA, the brains of the animals who were pre-administered with a sertraline vehicle, or sertraline alone, and in combination with the pro-convulsive agents, or LPS were removed, the hippocampus was dissected, placed in sterile tubes containing 1 ml of the TRIzol Reagent (Invitrogen Life Technologies, USA), and frozen at −80°C until used. Total RNA extraction was performed after hippocampus homogenization (15 strokes with a Thomas Scientific AA Teflon homogenizer) according to the manufactureŕs instructions. The RNA samples were suspended in 50 µl of nuclease-free water.

A nanodrop spectrophotometer (Thermo scientific, Wilmington, Delaware, USA) was used to determine the amount and purity of total RNA in each sample. The integrity of the total RNA was assessed by agarose gel electrophoresis, using ethidium bromide staining.

### cDNA Synthesis

The cDNA was obtained by reverse transcription of total RNA, using the kit SuperScript III First-Strand Synthesis SuperMix (Invitrogen Life Technologies, USA). For that purpose a small aliquot containing 2 µg of total RNA, suspended in nuclease-free water was mixed with 0.5 µl oligo (dT)_20_ (50 µM), 0.5 µl annealing buffer, and brought up to a final volume of 4 µl with nuclease-free water. This mixture was incubated at 65°C for 5 min and then chilled with ice. For reverse transcription, 1 µl of III/RNaseOUT™ enzyme mix and 5 µl of the 2X first-strand reaction mix (10 mM MgCl_2,_ and 1 mM of each dNTP) were added before incubation at 50°C for 50 min. The reaction was stopped by heating the mixture at 85°C for 5 min. The cDNA resulting from this procedure was stored at −20°C until use.

### Polymerase Chain Reaction (PCR)

The effect of sertraline on the rise of IL-1β and TNF-α mRNA expression induced by the convulsive agents was evaluated by reverse transcription-polymerase chain reaction (RT-PCR) using the kit GoTaq DNA Polymerase. Briefly, 1.5 µl of cDNA (250 ng/µl) were amplified in a mixture containing 2 µl of 5X green buffer, 0.8 µl of MgCl_2_ (25 mM), 0.25 µl of PCR nucleotide mix (10 mM), 0.5 µl of the sense primer (10 pM), 0.5 µl of the antisense primer (10 pM), 0.05 µl of DNA Polymerase (5 u/µl) and 4.4 µl of sterile Milli-Q water.

PCR reactions were done in an Eppendorf Mastercycler gradient (USA). The temperature cycling conditions were: initial denaturation at 95°C for 5 min, followed by 34 cycles, including denaturation at 94°C for 30 s, primer annealing for 45 s at 58°C for IL-1β and β-actin or at 66°C for TNF-α, and primer extension at 72°C for 1 min. A final primer extension was performed at 72°C for 10 min after which the samples were immediately cooled at 4°C. The nucleotide sequences for the IL-1β primers were: CCA-GGA-TGA-GGA-CCC-AAG-CA (sense) and TCC-CGA-CCA-TTG-CTG-TTT-CC (antisense); and the expected product size was 519 bp (gen bank accession NM_031512.2). The nucleotide sequences for the TNF-α primers were: AAG-CCC-GTA-GCC-CAC-GTC-GTA (sense) and GCC-CGC-AAT-CCA-GGC-CAC-TAC (antisense); and the expected product size was 663 bp (gen bank accession NM_012675.3). The nucleotide sequences for the β-actin primers were: ATC-GTG-GGC-CGC-CCT-AGG-CA (sense) and ACG-TAC-ATG-GCT-GGG-GTG-TTG (antisense); with the expected product size of 302 bp (gen bank accession NM_031144.2).

β-Actin was used as a control to normalize the relative mRNA amount of the amplified cytokines. A negative control in the absence of sample was run together with each transcript.

PCR products were separated by 1.5% agarose gel electrophoresis at 90 volts, stained with ethidium bromide and the resulting bands were quantified by densitometry using a MiniBIS Pro Gel Documentation System (Bio-America, Miami FL, USA) and ImageJ version 1.42 software (National Institute of Health, USA). Results are expressed as relative mRNA level expression (IL-1β/β-actin or TNF-α/β-actin ratio).

### Statistical Analysis

One-way analysis of variance (ANOVA) followed by a *post hoc* Tukey test was used for the statistical evaluations. Statistical analyses were performed with SigmaPlot version 11.0 (Systat Software, Germany). From *P*<0.05 the differences between data were considered statistically significant.

## Results

### Acute and Chronic Effect of Sertraline on IL-1β and TNF-α mRNA Expression in the Hippocampus

**Figure 2:**
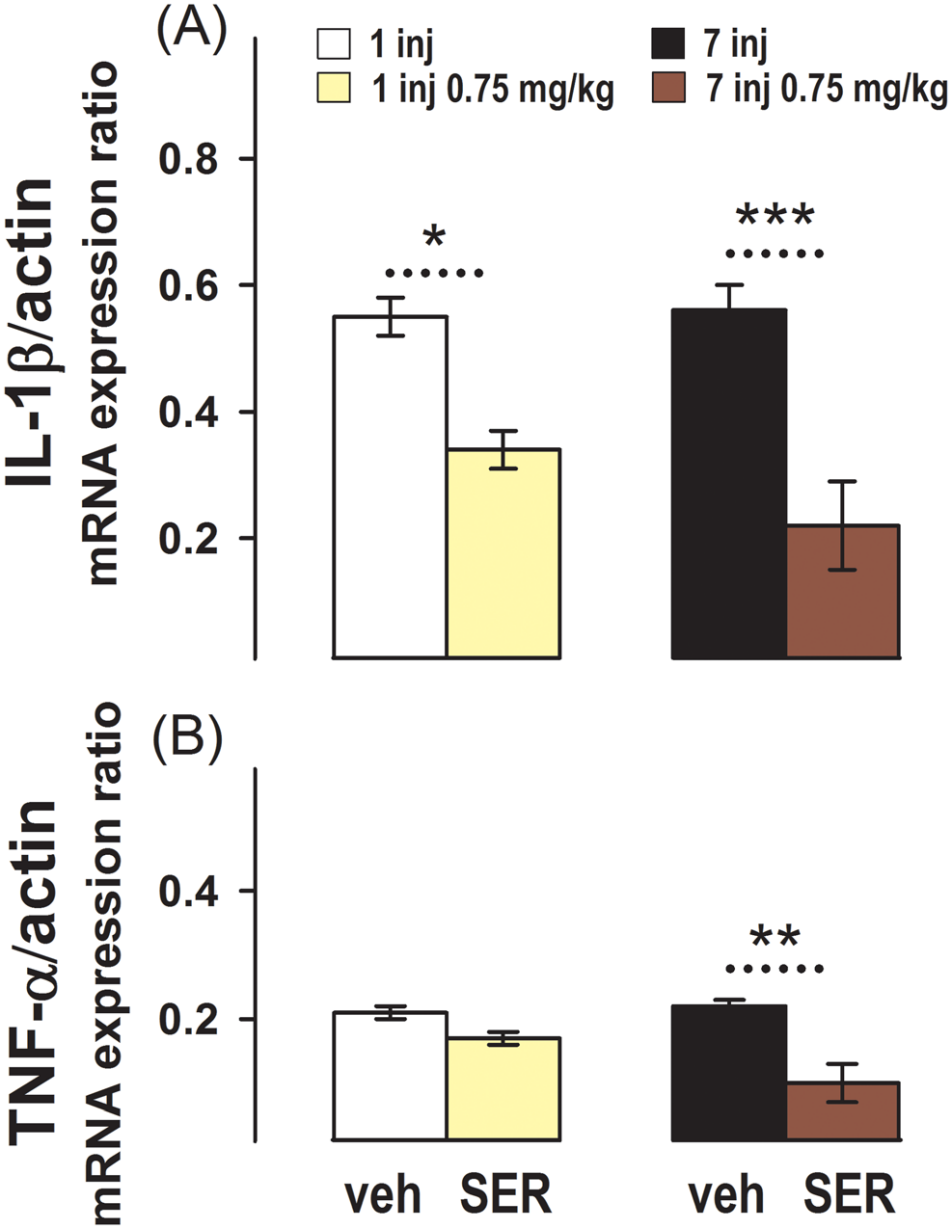
Effect of One or Several Sertraline Doses on Pro-inflammatory Cytokines Messengers Expression in the Hippocampus. Relative IL-1β/β-actin (**A**) and TNF-α/β-actin (**B**) mRNA expression measured by RT-PCR in the hippocampi of animals administered with (left bars): a single injection of vehicle (G1) or 0.75 mg/kg sertraline (G2); and in animals administered with (right bars): 7 injections for one week of vehicle (G3) or 0.75 mg/kg sertraline (G4). Results are the mean ± SEM values of 6 (G1 and G3), 4 (G2), or 3 (G4) animals per group. *, p = 0.01 and ***, p<0.001 between the dashed line connecting experimental conditions.

The effect of sertraline injected once or daily for one week on IL-1β and TNF-α messenger expression in the hippocampus is shown in Fig. 2. This figure shows that in the control groups injected with the vehicle once (G1), or 7 times (G3), the baseline mRNA expression of both cytokines is very similar.

In the animals administered once (G2), or for 7 days (G4) with sertraline 0.75 mg/kg, the expression of IL-1β mRNA was lower than in the respective controls injected once or for 7 days with the vehicle (Fig. 2A).

The expression of TNF-α mRNA was similar in animals injected once with the vehicle (G1), or with 0.75 mg/kg sertraline (G2). Although, in the animals injected daily with 0.75 mg/kg sertraline for one week the TNF-α messenger expression was again lower than in the control animals injected for 7 days with vehicle (Fig. 2B).

No difference between the expression of IL-1β and TNF-α mRNA in the hippocampus from rats administered with saline, or with the vehicle used to dissolve sertraline, namely 70% saline and 30% DMSO, was found. For instance IL-1β expression in animals injected once with saline and with 70% saline and 30% DMSO was 0.57±0.05 (mean ± SEM) and 0.53±0.05, respectively; and in animals injected for 7 days with saline and saline 70%/DMSO 30%, 0.60±0.06 and 0.52±0.05, respectively. In the case of TNF-α mRNA expression in animals injected once with saline, and with the sertraline vehicle was 0.20±0.01 and 0.23±0.01, respectively; and in the animals injected for 7 days, 0.21±0.01 and 0.22±0.02, respectively. Thus, all control animals, namely those injected with saline or the sertraline vehicle, were pooled and referred as “vehicle” in the figures.

### Effect of Acute and Chronic Sertraline at a Low Dose on Seizures Induced by 4-AP

Administering 4-AP *i.p.* at the dose of 2.5 mg/kg 4 h following the injection of the sertraline vehicle induced clonic-tonic seizures with limb extensions in all the rats of Group 5 (G5). The latency and duration of the first tonic-clonic seizure induced by 2.5 mg/kg 4-AP is shown in the first row of Table 2. In the group pre-administered with a single sertraline dose of 0.75 mg/kg 4 h before 4-AP (G6), the latency and duration of the first tonic-clonic seizure induced by 4-AP was similar to the control animals administered with vehicle before 4-AP (second row of Table 2). However, in G7, the group pre-administered daily with 0.75 mg/kg sertraline for one week the administration of 4-AP was unable to induce tonic-clonic seizures at all (bottom row in Table 2).

**Table 2:** Effect of One and Seven Sertraline Low Doses on 4-AP-Induced Seizures.

AnimalGroup	AdministeredSubstances	Animalsper Group	Latency § to the 1stTonic-Clonic Seizure	Duration § of the 1stTonic-Clonic Seizure	% of Rats PresentingSeizures
**G5**	**Vehicle** followed by **4-AP**	(6)	20.5±2.0	1.2±0.2	100
**G6**	**One Sertraline** 0.75 mg/kg Dose followed by **4-AP**	(4)	20.5±2.0	0.9±0.2	100
**G7**	**Seven Sertraline** 0.75 mg/kg Daily Doses followed by **4-AP**	(4)	NO Tonic-Clonic Seizures	NO Tonic-Clonic Seizures	0

§ *in min.*

*4-AP = 2.5 mg/kg 4-aminopyridine.*

*Results are the mean ± SEM values of the indicated number of animals.*

No conspicuous behavioral changes were observed in the groups administered with vehicle once (G1) or 7 times (G3), or with 0.75 mg/kg sertraline once (G2) or 7 times (G4). These groups were not included in Table 2, but the hippocampal mRNA expression of the pro-inflammatory cytokines in these groups was determined in parallel with the groups exposed to the convulsive agent.

### Effect of Sertraline on the Increase in IL-1β and TNF-α mRNA Expression Induced by 4-AP in the Hippocampus

**Figure 3:**
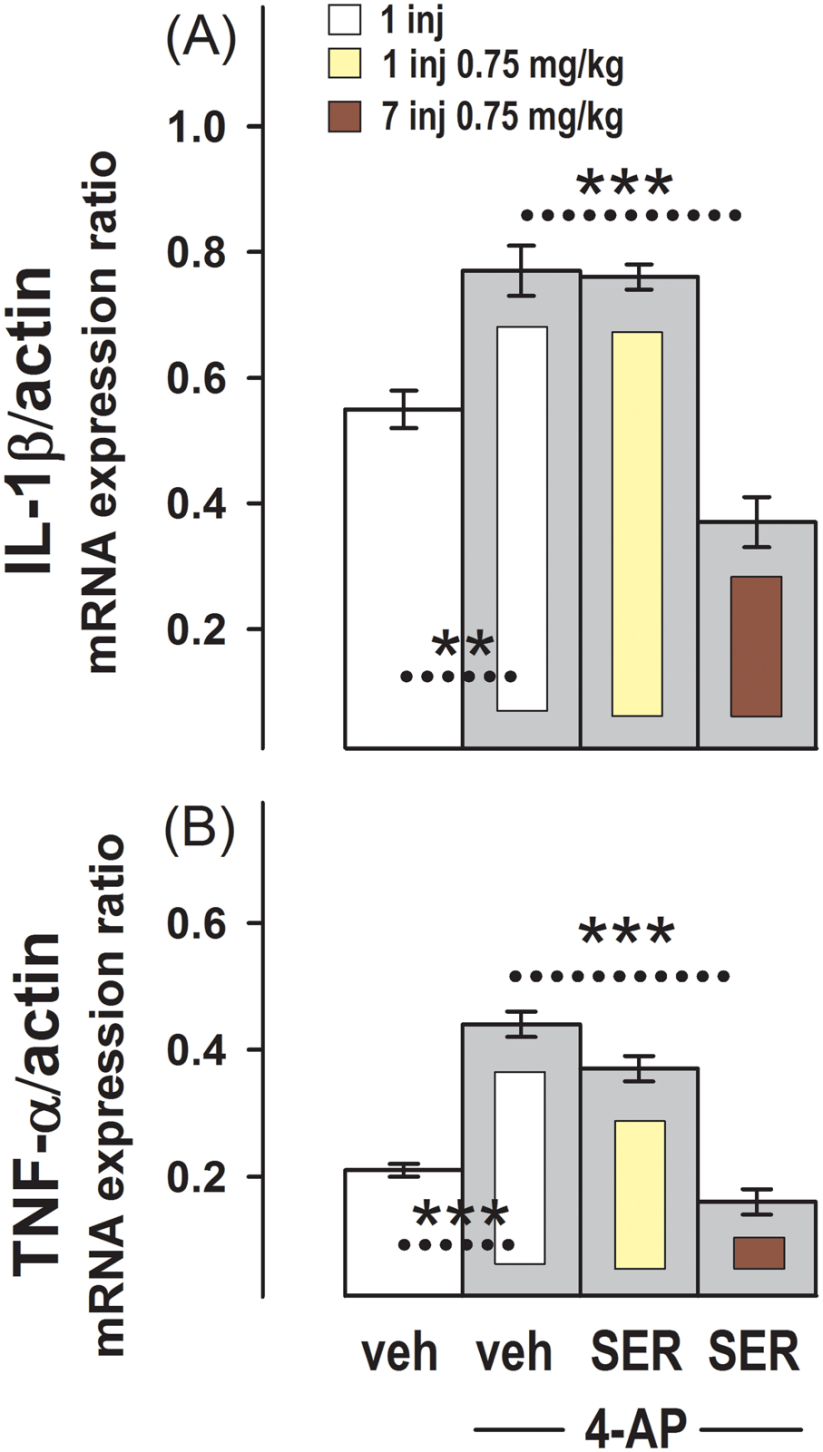
Effect of One or Several Sertraline Doses on the Increase in IL-1β and TNF-α mRNA Expression Induced by 4-AP in the Hippocampus. Relative IL-1β/β-actin (**A**) and TNF-α/β-actin (**B**) mRNA expression in the groups administered with a single injection of vehicle (G1) or with one daily injection of vehicle for one week (G3), and in groups injected with 2.5 mg/kg 4-AP and pre-administered with: a single injection of vehicle (G5), a single injection of 0.75 mg/kg sertraline (G6), or one daily injection of 0.75 mg/kg sertraline for one week (G7). Results are the mean ± SEM values of 6 (G5) or 4 (G6 and G7) animals per group.*, p = 0.01 and ***, p<0.001 between the dashed line connecting experimental conditions.

Fig 3 shows that in the group of animals administered with the convulsing agent, 4-AP (G5), the expression of IL-1β and TNF-α mRNA observed in the control group administered once with vehicle (G1) was increased. This increase induced by 4-AP on the expression of both cytokines was also observed in the group administered with the single dose of 0.75 mg/kg sertraline (G6). However, in the group administered for 7 consecutive days with 0.75 mg/kg sertraline (G7), 4-AP was unable to increase IL-1β or TNF-α mRNA expression above the control values observed in animals injected with vehicle.

### Effect of Sertraline on Seizures Induced by PTZ

The convulsive agent PTZ administered *i.p.* at the dose of 50 mg/kg 4 h after the injection of vehicle also induced clonic-tonic seizures with limb extensions in all the rats of Group 8 (G8). The latency and duration of the first tonic-clonic seizure induced by PTZ in this group is shown in the first row of Table 3. It is notable that 50 mg/kg PTZ induces the first tonic-clonic seizure very rapidly. A single injection of sertraline at a dose of 2.5 mg/kg 4 h before PTZ slightly increased the latency to the first tonic-clonic seizure induced by PTZ. At a dose of 2.5 mg/kg, however, sertraline was unable to prevent the tonic-clonic seizures induced by PTZ in most animals (second row in Table 3). The latency of this group (G9) was only assessed for the animals that had seizures. On the other hand, seizures induced by PTZ were completely suppressed in the group pre-administered with the single injection of 25 mg/kg sertraline 4 h before PTZ (third row in Table 3). No noticeable changes were observed for the 4 h after the single injection of 25 mg/kg sertraline.

**Table 3:** Effect of Sertraline at Two Doses on PTZ-Induced Seizures.

Animal Group	AdministeredSubstances	Animals perGroup	Latency § to the 1stTonic-Clonic Seizure	Duration § of the 1stTonic-Clonic Seizure	% of Rats Presenting Seizures
**G8**	**Vehicle + PTZ**	(7)	1.5±0.2	1.2±0.2	100
**G9**	**Sertraline** 2.5 mg/kg + **PTZ**	(5)	3.2±0.6*	0.8±0.3	80
**G10**	**Sertraline** 25 mg/kg + **PTZ**	(5)	NO Tonic-Clonic Seizures	NO Tonic-Clonic Seizures	0

§ *in min.*

*PTZ = 50 mg/kg pentylenetetrazole.*

*Results are the mean ± SEM values of the indicated number of animals.*

*, P<0.05 between the animals pre-administered with vehicle and with 2.5 mg/kg sertraline before PTZ.

### Effect of the Convulsive Agent PTZ on Hippocampal IL-1β and TNF-α mRNA Expression Levels in the Vehicle and in the Sertraline Pre-administered Animals

**Figure 4:**
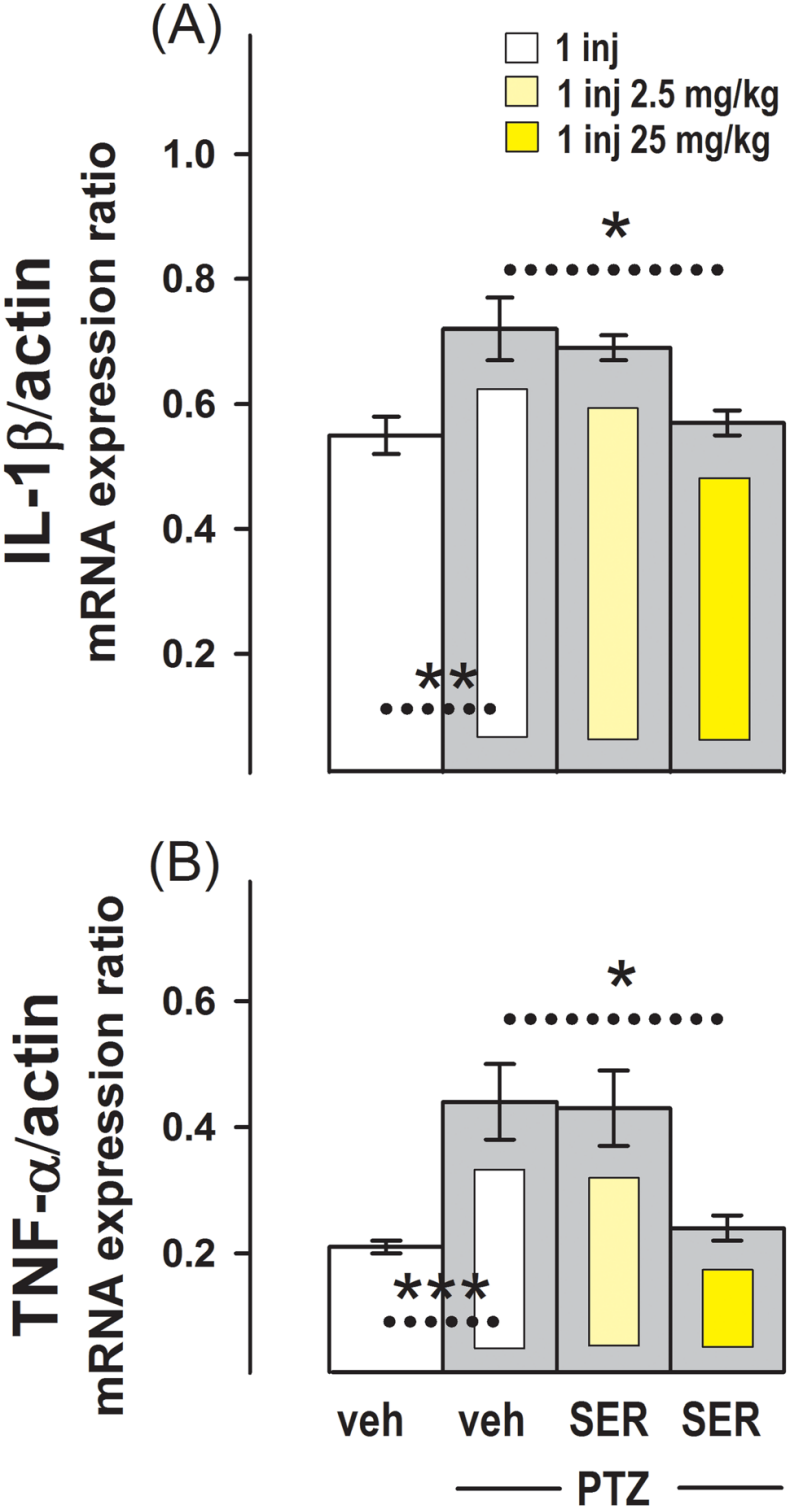
Effect of Sertraline on the Increase in IL-1β and TNF-α mRNA Expression Induced by PTZ in the Hippocampus. Relative IL-1β/β-actin (**A**) and TNF-α/β-actin (**B**) mRNA expression in the group administered with the single injection of vehicle (G1), and in groups injected with 50 mg/kg PTZ and pre-administered with single injections of: vehicle (G8), 2.5 mg/kg sertraline (G9), or 25 mg/kg sertraline (G10). Results are the mean ± SEM values of 7 (G8) or 5 (G9 and G10) animals per group. *, p<0.04; **, p<0.01 and ***, p<0.001 between the dashed line connecting experimental conditions.

Fig 4 shows that in the group of animals administered with PTZ (G8), the basal expression of IL-1β and TNF-α mRNA observed in the control group administered once with vehicle (G1) was increased. This increase induced by PTZ on the basal expression of both cytokines was also observed in the group pre-administered with sertraline at a dose of 2.5 mg/kg 4 h before PTZ (G9). However, in the group administered with 25 mg/kg of sertraline the typical increase in the IL-1β and TNF-α mRNA basal expression caused by PTZ was prevented.

### Effect of Sertraline on the Increase in IL-1β and TNF-α mRNA Basal Expression Induced by LPS

**Figure 5:**
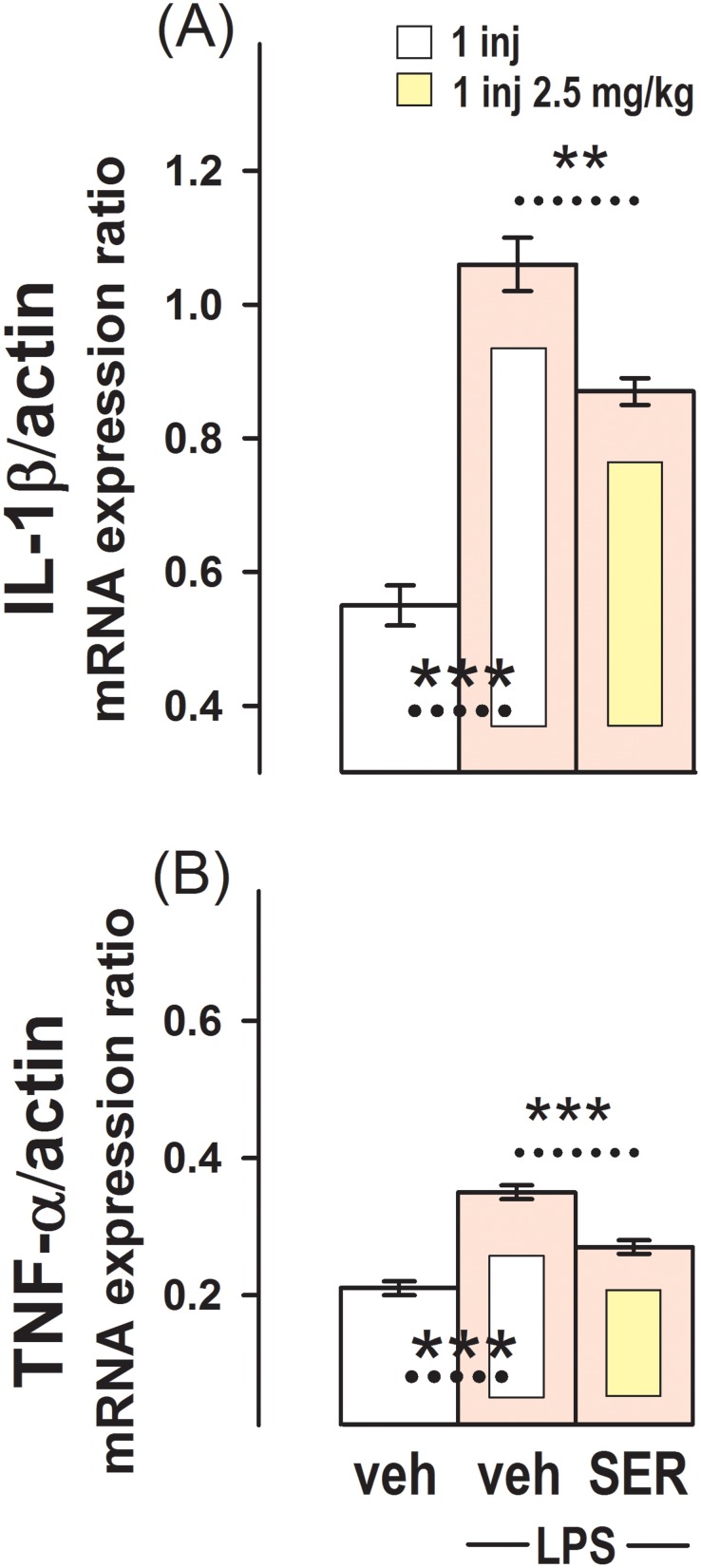
Effect of Sertraline on the Increase in IL-1β and TNF-α mRNA Expression Induced by LPS in the Hippocampus. Relative IL-1β/β-actin (**A**) and TNF-α/β-actin (**B**) mRNA expression in the group injected with vehicles (G1) and in groups inoculated with 100 µg/kg LPS and pre-administered vehicles (G11), or 2.5 mg/kg sertraline (G12). Results are the mean ± SEM values of 5 (G11) or 4 (G12) animals per group. **, p<0.01 and ***, p<0.001 between the dashed line connecting experimental conditions.

The effect of a single dose of 2.5 mg/kg sertraline on the rise in pro-inflammatory cytokines induced by LPS in the hippocampus is shown in Fig. 5. This figure shows that 1 h after the inoculation of LPS, at a dose of 100 µg/kg, the hippocampal mRNA expression of IL-1β and TNF-α in G11 was much higher than in the control group injected once with vehicle (G1). In the group pre-injected once with 2.5 mg/kg sertraline, the increase in the pro-inflammatory cytokines mRNA basal expression induced by the administration of LPS was less noticeable (G12).

## Discussion

In the present study the capability of the antidepressant sertraline to decrease the basal expression of the pro-inflammatory cytokines IL-1β and TNF-α from basal conditions in the hippocampus, was demonstrated. This finding along with the sensitivity to sertraline of the rise in IL-1β and TNF-α expression induced by LPS, strongly suggest a cerebral anti-inflammatory action of sertraline. In line there is evidence that pro-inflammatory cytokines can be modulated by some anti-depressants [27].

Mechanistic hypothesis of how immune-mediated changes and serotonin levels are involved in depression, as well as critical reviews of the effect of antidepressants on serotonin levels and inflammation, have been provided [28], [29]. However, our recent findings show that besides its action as a serotonin reuptake inhibitor, sertraline is an effective inhibitor of presynaptic Na^+^ channel permeability [22], and secretion of IL-1β and TNF-α in mixed glial cell cultures, was shown to be inhibited by the Na^+^ channel blockade [24]. Thus, another mechanistic explanation of how antidepressants, or at least sertraline, can affect inflammation is possible. For instance, it is probable that sertraline can reduce the baseline level of pro-inflammatory cytokines by decreasing the permeability Na^+^ channels controlling the release of inflammatory markers such as IL-1β and TNF-α.

Among the most effective antiepileptic drugs are those that reduce cerebral excitability, and stop the brain paroxysmal neuronal activity that accompany seizures, by blocking Na^+^ channels. Although, the potential anti-seizure capability of sertraline has not been explored before, probably because of reports suggesting that some selective serotonin reuptake inhibitors act as pro-convulsive drugs [30]. In fact, there used to be a black box warning in the Physicians Desk Reference (PDR) against their use in patients with epilepsy due to the possibility that they could reduce the seizure threshold, particularly in adolescents.

Although the selective serotonin reuptake inhibitor, sertraline, was needed in a higher dose to prevent the PTZ, than the 4-AP-induced changes, the capability of sertraline to prevent the increase of IL-1β and TNF-α expression, accompanying the generalized tonic-clonic seizures induced by both, 4-AP and PTZ, was also clearly demonstrated in the present study. This different efficacy might again be related with the capability of sertraline to directly decrease Na^+^ channel permeability [22], because the 4-AP mechanism of action involves several brain presynaptic ion channels, including Na^+^ channels [31], [32], but the PTZ mechanism of action is primarily due to a decrease in GABAergic transmission [33], [34].

The acute administration of 0.75 mg/kg sertraline to the rat reduced the expression of IL-1β in the hippocampus below basal conditions, but repeated doses were required to reduce TNF-α expression. It is amply recognized that both, anti-depressive and anti-epileptic drugs, are needed to be administered for a certain time before being able to produce their therapeutic action in patients. Administration of 0.75 mg/kg sertraline for several days was required to overcome the generalized tonic-clonic seizures induced by 4-AP, and the increase in IL-1β and TNF-α mRNA expression to 4-AP.

The single sertraline dose of 2.5 mg/kg given to the rat did not prevented the tonic-clonic seizures, and the rise in pro-inflammatory cytokines mRNA expression induced by PTZ, while the single sertraline dose of 25 mg/kg completely overcame the generalized tonic-clonic seizures as well as the increase in IL-1β and TNF-α mRNA expression induced by PTZ. In a previous study [25] we tested the effects of single sertraline doses in ranges from 2.5 to 25 mg/kg on the EEG epileptiform activity induced in the anaesthetized rat by PTZ, and found that a single sertraline dose of 5 mg/kg already prevented the epileptiform activity induced by PTZ in 75% of the animals, and single doses of 10, 15 and 20 mg/kg, completely prevented the EEG epileptiform activity induced by PTZ. Therefore, it is very possible that at doses below 25 mg/kg, sertraline will also be able to prevent the increase in IL-1β and TNF-α mRNA expression induced by PTZ. Moreover, bearing in mind that the higher effectiveness of sertraline after its repeated administration on the 4-AP induced changes, it might be expected that sertraline repeatedly administered will also prevent the increase in pro-inflammatory cytokines mRNA expression induced by PTZ at lower doses.

Several studies strongly suggest that brain pro-inflammatory cytokines may play a significant role in the generation and/or maintenance of seizures, and also in the establishment of chronic epileptic focuses [15], [17], [20]. Therefore, the putative brain anti-inflammatory action of sertraline suggested by present findings, might also contribute importantly to its anti-seizure effect.

Serotonin-reuptake inhibitors are known to decrease TNF-α and IL-1β serum levels in patients with major depressive disorders [35]. Seizures on the other side are known to increase BBB permeability [36]–[38]. The possibility that the drop in pro-inflammatory cytokines exerted by sertraline in the hippocampus was only due to its action on plasma levels, is unlikely. Present findings also show that sertraline decreases IL-1β and TNF-α expression from basal conditions in animals that were not exposed to the pro-convulsive agents, and did not experience seizures.

In summary, we conclude that the decrease in brain excitability and inflammation exerted by sertraline, makes this drug a highly potential alternative to control seizures.
